# Distribution and Behaviour of Some Trace Elements as a Function of Apple Varieties in Northeastern Romania

**DOI:** 10.3390/ijerph17072607

**Published:** 2020-04-10

**Authors:** Ionuţ-Mihai Prundeanu, Ciprian Chelariu, Sorin-Ionuț Balaban, Ovidiu-Gabriel Iancu

**Affiliations:** 1 Earth, Environmental and Life Sciences, University of Bucharest Research Institute (ICUB), 36–46 Mihail. Kogalniceanu Boulevard, 050107 Bucharest, Romania; prundeanu.ionut@gmail.com; 2 Department of Geology, University of Iaşi “Alexandru Ioan Cuza”, Carol I Boulevard, No. 20A, 700505 Iaşi, Romania; ogiancu@uaic.ro; 3 Department of Earth and Planetary Sciences, University of London, Birkbeck, Malet Street, London WC1E 7HX, UK; balabansorinionut@gmail.com

**Keywords:** trace elements, AAS, apple-orchard, apple varieties, Golden Delicious, Jonathan

## Abstract

The levels and distribution of 9 trace elements in apples from two orchards in north-east (NE) Romania were measured by means of Atomic Absorption Spectroscopy (AAS) on 42 samples of 9 different apple varieties, with average content ranges of 0.909–4.458 mg·kg^−1^ Zn, 0.055–0.409 mg·kg^−1^ Cu, 0.700–2.476 mg·kg^−1^ Fe, 0.328–0.695 mg·kg^−1^ Mn, 0.054–0.257 mg·kg^−1^ Ni, 0.005–0.101 mg·kg^−1^ Cr, 0.027–0.420 mg·kg^−1^ Co, 0.413–1.149 mg·kg^−1^ Pb, and 0.025–0.127 mg·kg^−1^ Cd. For some apple varieties, Pb contents are 2 times higher than the maximum contents allowed according to national standards, Cd contents are 6 times higher, and in some cases Zn contents also exceed the national threshold, showing preferential accumulation on specific apple varieties. Whilst some research has been carried out on trace element distribution in apples, this study assessed the areal distribution of toxic trace elements in connection to the adjacent roads. The first apple orchard is located near a county road, with reduced car traffic, while the second orchard shares its southern limit with a high-volume traffic road (E583). The results point towards a proportional increase of Pb and, to some extent, of Cd in the samples close to the E583 road in contrast with the other orchard, where no such observation derived. Along the areal distribution of the selected elements, the preferential accumulation of dietary recommended trace elements in different apple varieties was assessed. In matters of daily nutrients content in trace elements, the best sources of Fe, Cu, and Zn in terms of apple varieties are Golden Delicious, Jonathan, and Kaltherer Böhmer.

## 1. Introduction

The apple tree itself (*Malus pumila*) may be one of the earliest fruit trees ever cultivated by humankind [1]. Romania is the eighth-best apple-producing country in European Union and the 32nd worldwide, with a total output of 339,570.00 metric tons of apples, according to Food and Agriculture Organization statistics (FAO) [2].

Fruits are commonly regarded as one of the healthiest, well-balanced, and complex sources of nutrients for human diet. However, their quality is strongly related to their chemical composition. Hence, while some elements have a beneficial effect, there are others that, due to their dosage or bioaccumulation processes and chemical affinities, may become toxic. The chemical composition of food in general is of the utmost importance for consumers and health or nutrition specialists’ alike [3].

The latest years have witnessed a growing interest among medical and geoscientists alike in studying the contents of potentially toxic trace elements in plant-sourced foods as well as their distribution within the soil–plant and soil–fruit systems, aiming to properly trace and assess the abundance of toxic elements as well as the scarcity of healthy nutrients [4,5]. While some trace elements such as Cu and Zn play an important role in nutrition, others, such as Pb and Cd, may cause serious health issues, especially when they accumulate to dangerous levels within the plant or fruits, thus being listed as the most common pollutants in developing countries [6]. International databases and official reports [7] show a series of trace element concentrations for fresh fruit of 1.2 mg·kg^−1^ Fe, 0.4 mg·kg^−1^ Zn, and 0.27 mg·kg^−1^ Cu; for specific apple varieties such as Golden Delicious of 1.3 mg·kg^−1^ Fe, 0.4 mg·kg^−1^ Zn, and 0.30 mg·kg^−1^ Cu; and of 1.9 mg·kg^−1^ Fe for Jonathan and Jonagold in fresh weight with peel.

The chemistry of fruits may be influenced by a series of natural factors of a pedological or geological nature, especially when it comes to the soil substratum [8]. Other factors can be anthropic, such as pesticide usage, nearby industrial pollution sources, or road traffic [5,9]. Therefore, the chemical composition of plants will generally reflect the chemical composition of the environment in which they have grown [10].

Emissions of toxic trace elements from traffic activities is an important source of pollution to roadside ecosystems, such as orchards and farmlands [11]. This paper aims to investigate the potential influence of transportation activities on the toxic trace element concentrations in apples from two orchards located in Romania. To test this supposition, two orchards were selected, one near a low traffic load and the second orchard located near an important European road (E583). In addition, this research assessed the best apple varieties recommended for consumption when it comes to concentrations of dietary recommended trace elements, such as Fe, Cu, and Zn.

The list of chemical elements selected was based both on their toxicity and on the nutrient properties. Previous studies on apple composition from Romania focused mainly on the actual chemical concentrations of elements in the fruit [12,13,14], while this paper assesses the areal distribution of trace elements in apples grown near important motorway traffic routes and assesses if there is an impact on fruit chemistry.

## 2. Materials and Methods

### 2.1. Study Area

Fălticeni and Sârca are major apple-growing centres in northeastern Romania. Climatic conditions and geographical position of historical province of Moldavia facilitated the development of this region as one of the important apple-growing areas of Romania.

**Figure 1:**
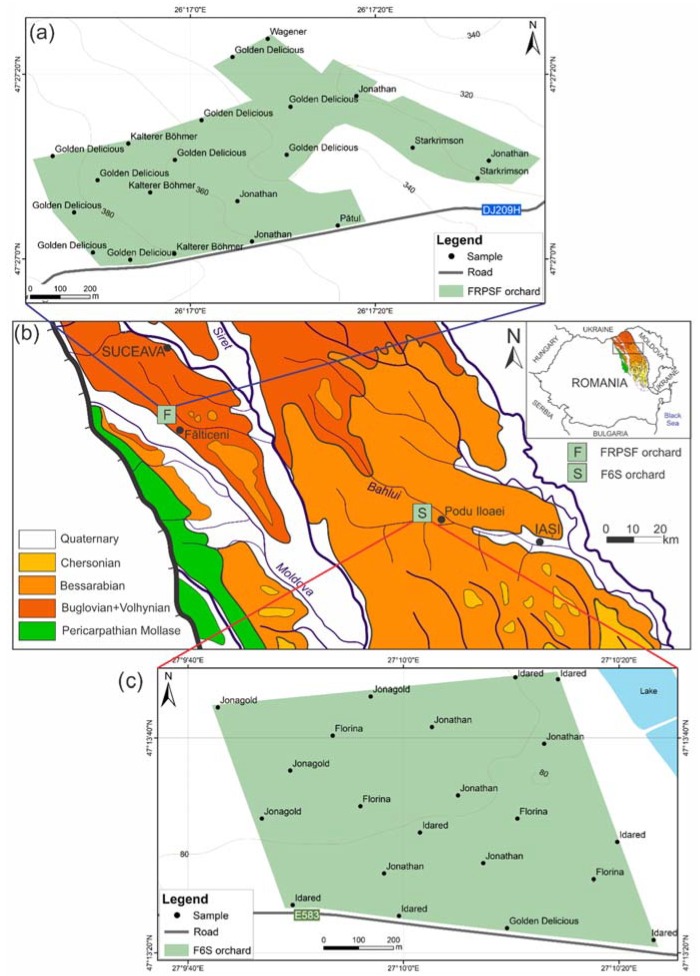
(**a**). Location of Fruit Research and Production Station of Fălticeni and the sampling network with apple varieties; (**b**). geological map of the Moldavian Platform with the location of the two studied areas (modified after Reference [18]); (**c**). location of Farm no.6 Sârca and the sampling network with apple varieties.

The soil’s detailed chemical composition and mineralogy for these two areas was previously investigated [15,16]. The first area belongs to the Fruit Research and Production Station of Fălticeni (FRPSF). The predominant soils are of Haplic Phaeozem and Rendzinic Phaeozem types, while the geological substratum consists of Volhynian deposits (Figure 1) of calcareous sandstones and oolitic limestone. The second area is farm no. 6 (F6S) from the Sârca orchards in south-east (SE) of the Jijia high-fields. The predominant soil types are Haplic and Calcareous-calcic Chernozems, while pelitic Bessarabian deposits of the Moldavian Platform (Figure 1) represent the geological substratum.

### 2.2. Samples and Sample Preparation

From the two studied areas, 42 samples (21 from each area (Figure 1)) covering nine apple varieties were collected at full fruit maturity, meaning commercial ripening. The sampling was performed at three days distance between the two orchards. The two orchards are of intensive growing type with conventional manuring. Two (Golden Delicious and Idared) of the nine apple varieties are among the most cultivated in Europe [17]. The total area of sampling was 40 hectares in the case of FRPSF and 50 hectares in the case of F6S. In order to pinpoint the location of each sample within the WGS84 coordinate system, a classic global positioning system (GPS) was used.

To avoid contamination, the sampling was done using polyethylene bags and polyethylene sealed boxes. The average mass of the samples was of 1000 g, which is six fruits per sample, all picked from the same tree. Fridges were used for storage. Subsequently, the apples were washed with distilled water and vertically sliced, removing the seeds. Approximately one third of the entire pooled sample was progressively dehydrated up to a temperature of 105 °C [13] over an 8 h cycle. In this step, the water content of the different apple varieties was determined. The samples were weighted before and after the dehydration. This step was carried out on the whole fruit, including the peel, without the seeds. The dried samples were then grounded, homogenized, and stored in polyethylene boxes.

### 2.3. Analytical Methods

In the step of sample preparation for trace elements content determinations, HCl 6N, HCl 0.5N were used, and Merck Certipur standards (Zn, Cu, Fe, Mn, Ni, Cr, Co, Pb, and Cd) and blank solutions were used in the calibration process. The results reported for this study are in mg·kg^−1^ of fresh fruit if not stated otherwise.

For this study, the contents of the 9 studied trace elements were measured through Atomic Absorption Spectroscopy (AAS), and the following laboratory equipment was used: electric calcination oven Nabertherm L9/11/B170 (Nabertherm GmbH, Lilienthal, Germany) with automatic time and temperature settings (maximum achievable temperature 1600 °C), SOLAAR S4 spectrometer (Thermo Fisher Scientific, Waltham, MA, USA) with automatic measurement logging, and hollow cathode lamps. The analyses were performed at the National Research and Development Institute for Soil Science, Agrochemistry, and Environment—ICPA Bucharest, Romania.

A quantity of 1 g of dry vegetal material was weighed for each sample and then burned on a Bunsen Burner, after which it was calcined in the oven at 450 °C for 6 h. The sample was then treated with 1 mL HCl 6N, and the HCl was evaporated on a sand-bath; then, the HCl 6N treatment was repeated. The samples were then passed into 25-mL volumetric flasks with HCl 0.5N to level.

The spectrometer was calibrated, and the standards were measured, after which the samples themselves were analysed. The results were converted to element concentration in fresh fruit.

The detection limits for the analysed elements are Zn = 0.005 mg/L, Cu = 0.02 mg/L, Mn = 0.01 mg/L, Pb = 0.10 mg/L, Ni = 0.04 mg/L, Co = 0.05 mg/L, Cd = 0.005 mg/L, Fe = 0.006 mg/L, and Cr = 0.005 mg/L.

Distribution maps were built in ArcGIS 10.5 Software (ESRI, Redlands, CA, USA) by using Inverse Distance Weighting (IDW) as a data interpolation method. Statistical parameters such as mean, geometric mean, median, first, third quartiles, standard deviation, coefficient of variation, skewness, and kurtosis were also calculated, and the average content of trace elements and water for each apple variety and geochemical correlations were determined.

## 3. Results and Discussion

### 3.1. Water Content

The water content of the fruits was measured on all 42 samples (Table 1). The samples from FRPSF had higher water content than the ones from F6S. The average water content for the apples of FRPSF is 3.25% higher compared to F6S. Similar variation has been noted for two varieties cultivated in both areas: Jonathan (4.85%) and Golden Delicious (3.74%).

The difference between the water contents of apples from the two areas may be a result of Baltic climate influences on the FRPSF compared to F6S. This variation consists mainly of higher precipitations. FRPSF is located approximately 85 km to the northwest of F6S. Generally, climatic conditions are an important factor in influencing the quality and chemical composition of fruit [19].

The average water content for all 42 samples is 84.46%, close to other values obtained for apples grown in Spain (81.9%), Italy (83.7%), Poland (84.8–89.9%), and The Netherlands (87.4%) [20,21].

### 3.2. Trace Elements Content

The average contents for the 9 studied trace elements for each variety are given in Table.

For FRPSF, important nutrients contents such as Zn and Fe were determined for the Golden Delicious as well as important contents of Cu for the Jonathan and Kaltherer Böhmer varieties. The Golden Delicious variety from F6S stands out through higher Zn and Pb contents than FRPSF as well as high Cu contents for the Idared, Florina, and Golden Delicious varieties.

In a study performed in NW Romania, where Cu was determined through inductively coupled plasma mass spectrometry ICP-MS [13], the values expressed in dry weight (d.w.) were similar to the ones presented in this paper for the Idared variety of 2.09 ± 0.78 mg·kg^−1^ Cu (1.48 mg·kg^−1^ Cu d.w. FRPSF) and significantly higher for the Golden Delicious variety of 6.14 ± 1.24 mg·kg^−1^ Cu (1.45 mg·kg^−1^ Cu d.w. FRPSF and 1.53 mg·kg^−1^ Cu d.w. F6S). This difference in case of Golden Delicious variety is suggested to be sourced in the use of Cu-based fungicides, fertilizers, and the different soil type/geological background.

For the two studied areas, Pb and Cd exceed the maximum content thresholds specified by Romanian national legislation [22,23] for fresh fruit in seven out of the nine analysed varieties. At FRPSF, the national Pb content thresholds are exceeded in 10 samples from 4 varieties (Golden Delicious, Starkrimson, and Jonathan, Pătul) while, for Cd, this threshold is exceeded in 19 samples from 21, five varieties being impacted, Wagener not included. As for the F6S, the Pb threshold is exceeded in 18 samples from all five varieties while the Cd threshold is exceeded in 17 samples from all varieties with the exception of Golden Delicious.

**Figure 2:**
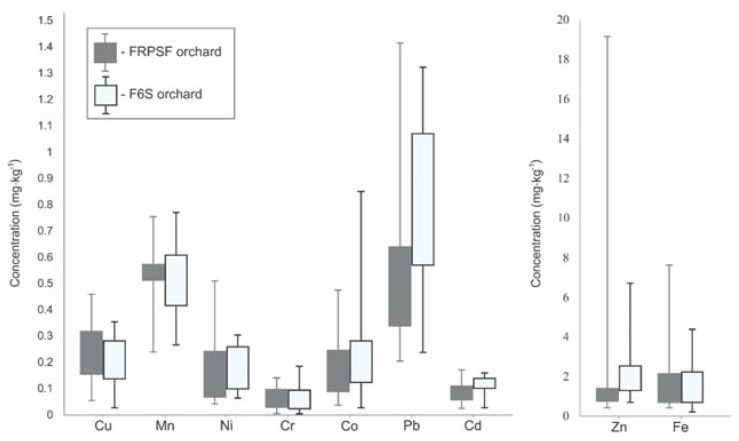
Boxplot with the concentrations of trace elements in samples from both orchards: The central rectangle represents the range between the first quartile and the third quartile, while the whiskers above and below the rectangle show the values of the minimum and maximum. FRPSF—Fruit Research and Production Station of Fălticeni; F6S—Farm no.6 Sârca.

The contents of Fe, Zn, and Cu are also important as they can bring an important intake of nutrients in human daily diet. The most balanced from this point of view is the Jonathan variety, which has similar Zn and Fe contents in both studied areas. The average Zn content for the FRPSF is not representative for the dataset, mainly due to the interference caused by the very high content determined for one single sample (19.181 mg·kg^−1^ Zn); therefore a more fit parameter would be the median (1.223 mg·kg^−1^) (Figure 2). The same is valid for Fe content.

In the FRPSF orchard for Ni (4 samples), Cr (4 samples), and Co (5 samples), the contents were below detection limit while, at F6S, the contents of Ni (3 samples), Cr (7 samples), Co (4 samples), and Cu (2 samples) were below detection limit.

Correlation matrices aim to identify the similarities and competition between bioaccumulation processes of some elements (Table 2). With the exception of a positive correlation between Fe and Ni, and a negative correlation for Cd–Co (both from FRPSF), there were no significant correlations identified in the two studied areas. The Fe–Ni correlation can be explained by the fact that Ni has a siderophile affinity, therefore easily becoming associated with Fe [24].

#### 3.2.1. Zinc (Zn)

The presence of organic matter and clay minerals in soils gives them a high capacity of immobilizing Zn, especially in neutral to alkaline pH conditions [10]. When it comes to absorption by plants, Zn competes with Fe and the relationship with Cd may be antagonistic and synergetic at the same time [24].

**Figure 3:**
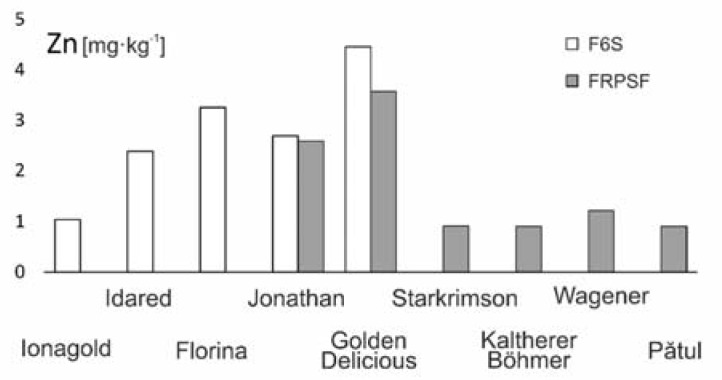
Zn content variation according to apple varieties in FRPSF and F6S orchards. FRPSF—Fruit Research and Production Station of Fălticeni; F6S—Farm no.6 Sârca.

The average Zn content in the analysed samples from both sites varied in this order: Golden D. (Sârca) > Golden D. (Fălticeni) > Florina > Jonathan (Sârca) > Jonathan (Fălticeni) > Idared > Wagener > Jonagold > Starkrimson > Kaltherer B. > Pătul (Figure 3). In a study on 10 apple varieties from northwestern Romania [13], high Zn contents were obtained for the Golden D. and Florina varieties, the Zn abundance order for common soils being Golden D. > Starkrimson > Florina > Jonathan > Idared > Jonagold. Similar values were obtained for the Golden D. (4.2 mg·kg^−1^) cultivated in Muntenia (Romania) [25]. Suggesting that the Golden D. variety has an enhanced capacity of assimilating Zn, this can be observed from Figure 3. This places the Golden D. variety as a good source of Zn; the recommended daily intake is 9.5 mg/day for men and 7.0 mg/day for women [26].

Other studies from countries with a tradition in apple trees growing, such as Poland [27,28], present lower Zn values in fresh apples compared to the current paper, while studies from Serbia show similar Zn concentrations [29] (Table 3).

#### 3.2.2. Copper (Cu)

The repeated and sustained use of fertilizers over long periods can lead to important Cu accumulations in the upper soil horizon [30]. Another anthropic Cu source is brake wear from transportation, while the bitumen and other mineral filler materials in asphalt road surfaces contain heavy metals like Cu, Zn, Pb, and Cd [31]. Cu deficiencies tend to show on acidic sandy soils and occasionally in alluvial soils with high humus content [32]. Cu is indispensable to plant development, fulfilling various vital roles such as respiration, photosynthesis, and metabolism [24]. Copper is important in human nutrition, and the average ingested quantity for adults varies between 1 and 2.5 mg daily, while the European population reference intake is 1.1 mg/day for adults [26]. However, lack of copper causes anemia, asthenic mood, difficulty in breathing, and skin ulcerations.

**Figure 4:**
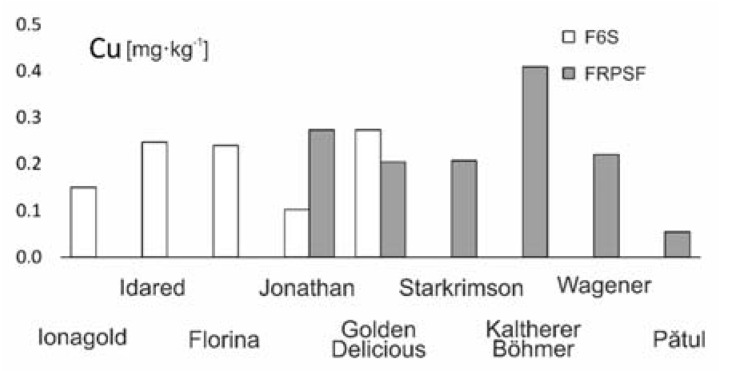
Cu content variation according to apple varieties in FRPSF and F6S orchards. FRPSF—Fruit Research and Production Station of Fălticeni; F6S—Farm no.6 Sârca.

**Figure 5:**
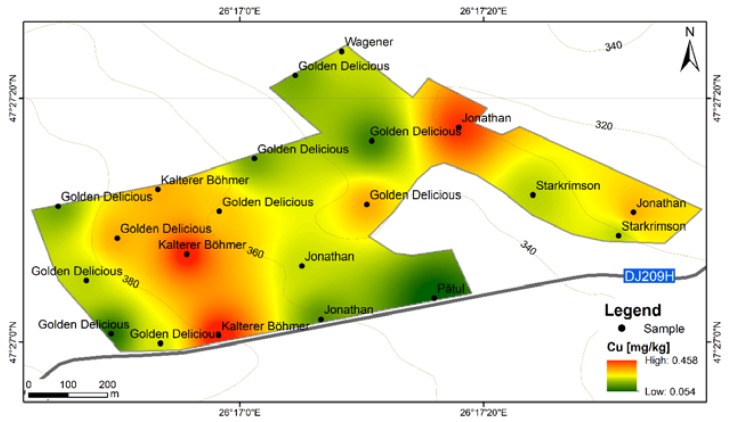
Cu content distribution according to apple varieties in FRPSF orchard. FRPSF—Fruit Research and Production Station of Fălticeni; F6S—Farm no.6 Sârca.

Average Cu contents from both areas are similar (Figure 4) (0.240 mg·kg^−1^ for FRPSF and 0.201 mg·kg^−1^ for F6S); however, for some samples from F6S, the Cu content was below detection limit. The high Cu values for the FRPSF is due to the Kaltherer B. variety (Figure 5), which may have a better Cu accumulation capacity. The other varieties have closely similar values of Cu contents, and there are no notable differences.

The order of abundance given by average Cu contents from apples from both studied sites is Kaltherer B. > Golden D. (Sârca) > Jonathan (Fălticeni) > Idared > Florina > Wagener > Starkrimson > Golden D. (Fălticeni) > Jonagold > Jonathan (Sârca) > Pătul (Figure 4). This Cu abundance order presented in another study [13] (Golden D. > Jonathan > Florina > Jonagold > Starkrimson > Idared) is different, but generally, Cu values are similar to the ones in the current study. Other studies [14] indicate higher Cu contents for fresh apples grown locally in Romania as well as imported ones (Table 3).

#### 3.2.3. Iron (Fe)

In soils, it comes mainly from natural sources but may also occur from herbicides and fertilizers.

**Figure 6:**
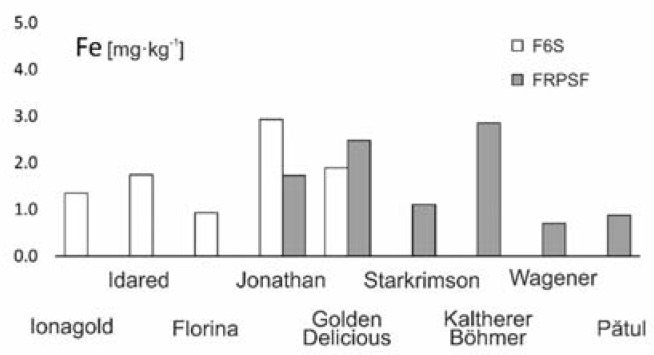
Fe content variation according to apple varieties in FRPSF and F6S orchards. FRPSF—Fruit Research and Production Station of Fălticeni; F6S—Farm no.6 Sârca.

For the two areas, the abundance order given by the average Fe contents is Jonathan (Sârca) > Kaltherer B. > Golden D. (Fălticeni) > Golden D. (Sârca) > Idared > Jonathan (Fălticeni) > Jonagold > Starkrimson > Florina > Pătul > Wagener. For the Jonathan (northeastern part of the Sârca orchard) and for Kaltherer B. and Golden D. (western part of the FRPSF), the average contents are over 2 mg·kg^−1^ in fresh fruit (Figure 6). The abundance order of Fe in a similar study [13] is Jonathan > Golden D. > Starkrimson > Jonagold > Idared > Florina, also with higher contents of Fe for Jonathan and Golden D. than in other varieties. The recommended Fe dietary intake is estimated to be between 8 and 10 mg/day [26].

When it comes to the Kaltherer B. variety, we did not find recent studies focused on the chemical composition. This is most probably because it already is a “classic” variety [42]; it is no longer cultivated on a large scale, mainly due to the climatic conditions that are necessary for this variety to thrive. Other studies show similar results for the orchard of Voinești, Romania [12] (Table 3), while for apples cultivated in the Balkans, Fe appears as a dominant trace element [43].

There was no notable correlation between Fe contents in fruits and the ones obtained in other study [16] for the topsoil from the same two areas.

#### 3.2.4. Cadmium (Cd)

Cd is one of the most eco-toxic metals, with similar behaviour to Zn [24]. In soils, important Cd accumulations can be sourced in the phosphatic fertilizer use [44]. From transportation, the largest emissions are due to engine oil consumption and subsequently sourced in the wear of asphalt cover of roads made by bitumen and other filling materials [31].

**Figure 7:**
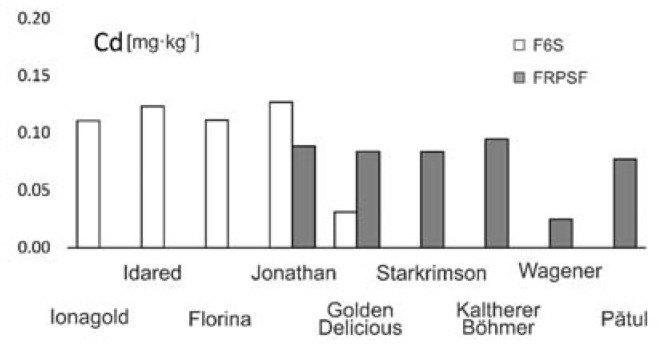
Cd content variation according to apple varieties in FRPSF and F6S orchards. FRPSF—Fruit Research and Production Station of Fălticeni; F6S—Farm no.6 Sârca.

**Figure 8:**
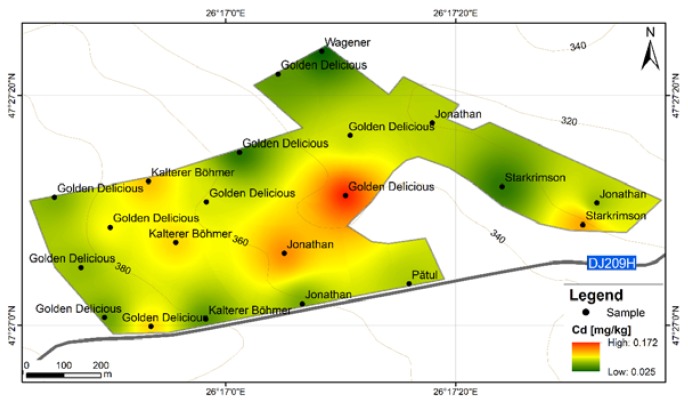
Cd content distribution according to apple varieties in FRPSF orchard. FRPSF—Fruit Research and Production Station of Fălticeni; F6S—Farm no.6 Sârca.

In the present study, this toxic element exceeds the maximum admitted content limit for 36 out of 42 samples and for 7 out of 9 apple varieties from both orchards. The order of Cd abundance given by average contents is Jonathan (Sârca) > Idared > Florina > Jonagold > Kaltherer B. > Jonathan (Fălticeni) > Golden D. (Fălticeni) > Starkrimson > Pătul > Golden D. (Sârca) > Wagener (Figure 7). The spatial distribution of Cd in apples from Fălticeni is uniform, with a slight increase towards the central part of the FRPSF (Figure 8).

**Figure 9:**
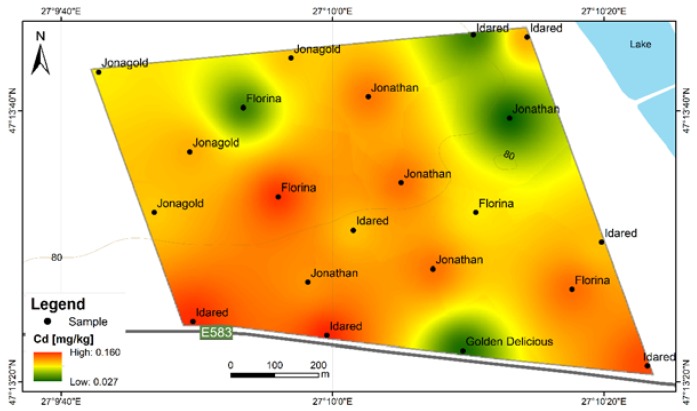
Cd content distribution according to apple varieties in F6S orchard; F6S—Farm no.6 Sârca.

For the Sârca orchard, Cd contents show an increase for the samples taken from the vicinity of the main road (Figure 9). Without clear evidence as samples, more distant to the traffic source are still showing high values of Cd. This aspect might suggest a Cd accumulation due to road traffic by suspended road dust caused by studded tires and road salt but without obvious proof from the distribution map. There is an exception for the Golden D.; however, these varieties represent the youngest trees from the orchard, which may explain the difference by the lower exposure and points out that the same is not observed for the Fălticeni orchard. The samples near the road vicinity do not indicate a preferential distribution as in the case of F6S. This could be sourced in the different traffic volumes of the two roads; the one at F6S is a European road (E583), while the one at the border of FRPSF is a county road (DJ209H).

The authors could not find any other relevant studies that investigate the Cd abundance in apple varieties. In other papers [13], the reported values are below detection limit (<0.01 mg·kg^−1^) for all varieties, while other studies did not show more than 0.04 mg·kg^−1^ for both locally grown and imported varieties [14]. Lower Cd values were obtained for the Starking variety in Portugal [37] (Table 3).

#### 3.2.5. Lead (Pb)

In soils, it accumulates in clay fractions and it is absorbed by Fe and Mn hydroxides. There is no known function that Pb fulfils in plant metabolism [10], but it is well worth mentioning due to its’ toxicity. An important source of antrophic Pb for the two orchards in this study is the car traffic from the vicinity roads. Brake wear is the most significant source of Pb from car emissions. Though the introduction of unleaded gasoline has considerable decreased the emissions of Pb, it may still be present in the exhaust gas being sourced in the worn metal alloys in the engine [31].

**Figure 10:**
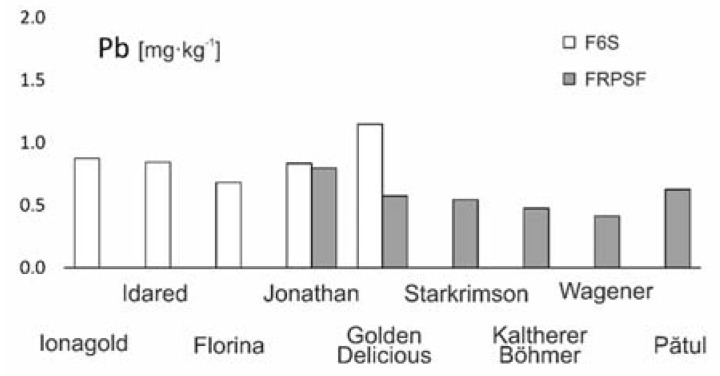
Pb content variation according to apple varieties in FRPSF and F6S orchards. FRPSF—Fruit Research and Production Station of Fălticeni; F6S—Farm no.6 Sârca.

**Figure 11:**
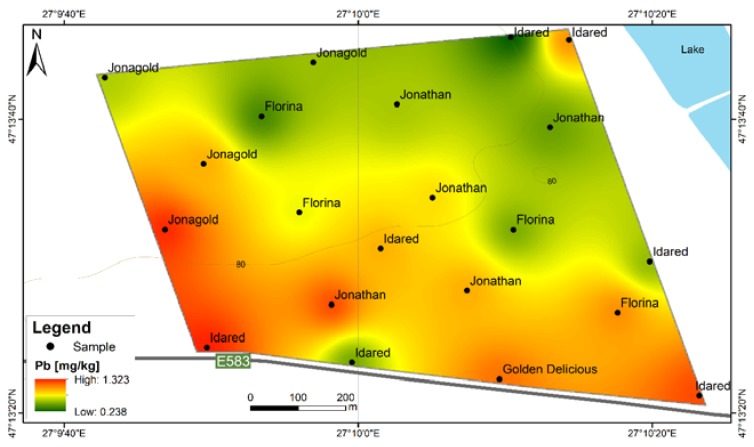
Pb content distribution according to apple varieties in F6S orchard; F6S—Farm no.6 Sârca.

The abundance of this element in apples from both areas varies in this order: Golden D. (Sârca) > Jonagold > Idared > Jonathan (Fălticeni) > Jonathan (Sârca) > Florina > Pătul > Golden D. (Fălticeni) > Starkrimson > Kaltherer B. > Wagener (Figure 10). The average Pb content for both orchards (0.714 mg·kg^−1^ in fresh apples) is over the national allowed threshold (of 0.5 mg·kg^−1^), with Pb average values of 0.595 mg·kg^−1^ for FRPSF and 0.832 mg·kg^−1^ for F6S (Figure 11). All apple varieties from Sârca have average values that exceed the maximum threshold. The only two varieties with Pb below 0.5 mg·kg^−1^ are Wagener and Kaltherer B. from Fălticeni orchard.

Beside one Idared sample, a strip of Pb concentrations at F6S shows a proportionally decrease with distance from the road, indicating, with more confidence than in the case of Cd, a possible source of accumulation in fruits due to the vicinity of high-volume traffic roads. In comparison, this behavior is not present on the distribution map of Pb at FRPSF. The Fălticeni orchard being located next to a county road is in contrast to F6S, which is placed next to a high-traffic European road (E583).

**Figure 12:**
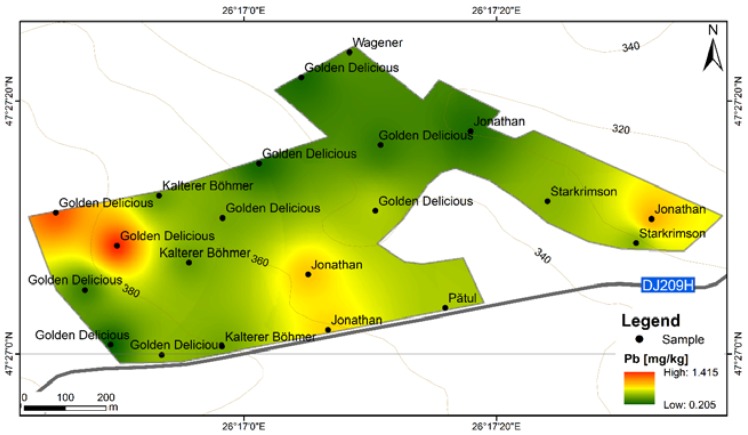
Pb content distribution according to apple varieties in FRPSF orchard; FRPSF—Fruit Research and Production Station of Fălticeni.

The lowest values for Pb contents were obtained for the Wagener variety from Fălticeni (0.413 mg·kg^−1^.); it is worth mentioning that this sample is located the farthest distance from the road, being at least exposed to car traffic influence. For this orchard, the Pb contents are close to national legislation limits, and the highest content is found to be at the borderline between the orchard and a private property (Figure 12).

Other studies reported lower values (Table 3) for apples from Poland [27,28] or Turkey [39] but also in Romania [14]. A comparison regarding the abundance variation order versus the variety of apple was not possible here, as no other study concerning this aspect for lead was found. Moreover, in a study on apples from NW Romania, the Pb values are below detection limit for all varieties [13].

#### 3.2.6. Manganese (Mn)

Mn is a most vital element in plant nutrition, and scarcities of Mn are often associated with Zn deficiencies [32]. For the two studied orchards, Mn presents a distribution that is similar to that of Cu and Fe.

The average values (0.546 mg·kg^−1^ at FRPSF and 0.516 at F6S) match closely with similar studies done in Greece [34] and U.S.A. [41], reported for the dry mass (Table 3). There have been lower contents found in Portugal [36] and Poland [28].

**Figure 13:**
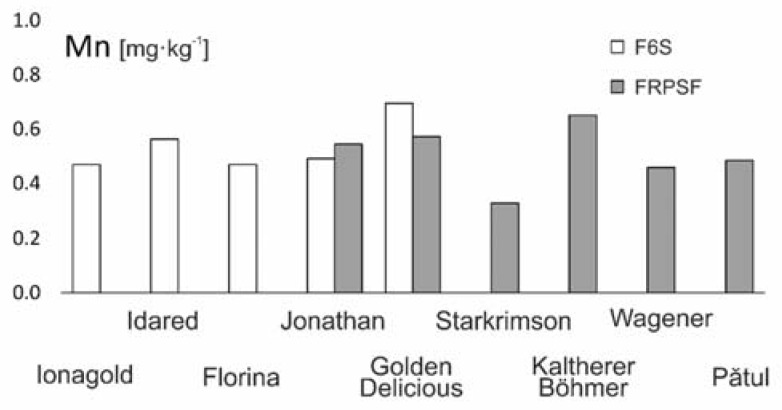
Mn content variation according to apple varieties in FRPSF and F6S orchards. FRPSF—Fruit Research and Production Station of Fălticeni; F6S—Farm no.6 Sârca.

The order of abundance given by the average Mn contents for both orchards is Golden D. (Sârca) > Kaltherer B. > Golden D. (Fălticeni) > Idared > Jonathan (Fălticeni) > Jonathan (Sârca) > Pătul > Florina > Jonagold > Wagener > Starkrimson (Figure 13). The higher Mn content in Golden D. present in both areas does not direct any strong suggestion as, opposite to this observation, a similar study reported a different order (Jonagold > Idared > Jonathan > Starkrimson > Florina > Golden D.) in which the Golden D. had the lowest Mn content [13].

#### 3.2.7. Nickel (Ni)

Ni plays an important role in biological systems [39], and the bioavailability for plants is dependent on its origin and the soil characteristics, in which the pH has a crucial role, as well as on the plants’ own abilities to absorb Fe. The interaction between Fe and Ni is apparently a common mechanism that is involved in Ni toxicity [24].

The dependency between Ni content from apples and some of the soils’ characteristics (quantity of bioavailable Mn, K, and humus proportion in the soil fraction <0.002 mm) was noticed [27]. Variations of Ni concentrations depending of soil characteristics were noticed. For example, at F6S, where the soil is a chernozem cambic and calcaric, we found higher contents than at FRPSF, where the soil is of phaeozem, haplic, and rendzina types. For the same apple varieties, Jonathan and Golden Delicious, the Ni concentrations are higher at F6S. The same is valid for Ni concentrations in the soil of the two orchards [16], suggesting that a higher background concentration in soil would lead to an increased accumulation in fruits considering the same apple varieties.

**Figure 14:**
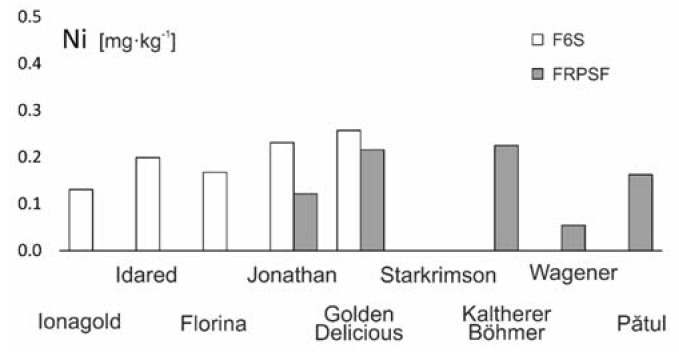
Ni content variation according to apple varieties in FRPSF and F6S orchards. FRPSF—Fruit Research and Production Station of Fălticeni; F6S—Farm no.6 Sârca.

The Ni distribution order given by the average contents is Golden D. (Sârca) > Jonathan (Sârca) > Kaltherer B. > Golden D. (Fălticeni) > Idared > Florina > Pătul > Jonagold > Jonathan (Fălticeni) > Wagener > Starkrimson (Figure 14).

A higher capacity of Golden D. to absorb Ni for both orchards is noticed, while for the Jonathan variety, the Ni contents are at a half level. The distribution of Ni follows the one of Fe; as a result, their geochemical behavior of the two elements is similar. There are no obvious influences of road traffic closeness on the Ni’s spatial distribution.

Similar Ni contents were reported for apples from Serbia [29,38] and Greece [5], while higher contents were found close to mining sites [9] (Table 3).

#### 3.2.8. Chromium (Cr)

The main Cr source in soils is the preexisting geological substratum from which they have developed. Cr has a low mobility, and it is very dependent on soil conditions. In neutral soil pH, it is not available to plants [10]. To present date, there is no clear evidence regarding the role of Cr in plant metabolism [24].

At Sârca, Cr has a similar distribution to that of Cu, with some samples where the content is below detection limit. Other authors [40] have obtained similar values for organic apples from Turkey (Table 3). At Fălticeni, higher Cr contents are encountered for the Jonathan and Kaltherer B. varieties.

The general Cr distribution order given by the average contents is Jonathan (Fălticeni) > Jonathan (Sârca) > Idared > Kaltherer B. > Starkrimson > Wagener > Golden D. (Fălticeni) > Florina > Jonagold > Pătul > Golden D. (Sârca), with the mention that 11 of 42 samples having results below the detection limit (<0.005 mg·kg^−1^). Values below detection limit (<0.10 mg·kg^−1^) were reported in other studies [13].

**Figure 15:**
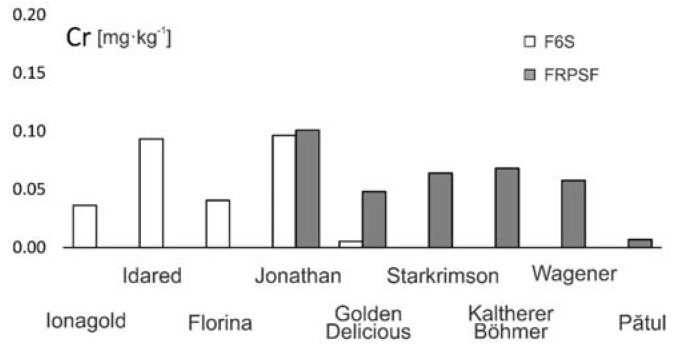
Cr content variation according to apple varieties in FRPSF and F6S orchards. FRPSF—Fruit Research and Production Station of Fălticeni; F6S—Farm no.6 Sârca.

The highest Cr contents from both areas are reported for the Jonathan and Idared varieties (Figure 15), which may point out a higher bioaccumulation capacity for these. The lowest contents were measured for the Golden D. and Pătul varieties.

Similar values were encountered in other studies for apples grown in Greece [5], where the authors indicate the geological substratum and fertilizer as Cr sources.

#### 3.2.9. Cobalt (Co)

Co presence in soils is highly influenced by the formation of manganese oxides, which have a high absorption capacity. Within the upper soil horizon, there are notable correlations of Co with other trace elements [45]. The main Co source is geogene, but high contents were reported near motorways and roads. One of the important roles of Co is in the stimulation of chlorophyll formation [23].

**Figure 16:**
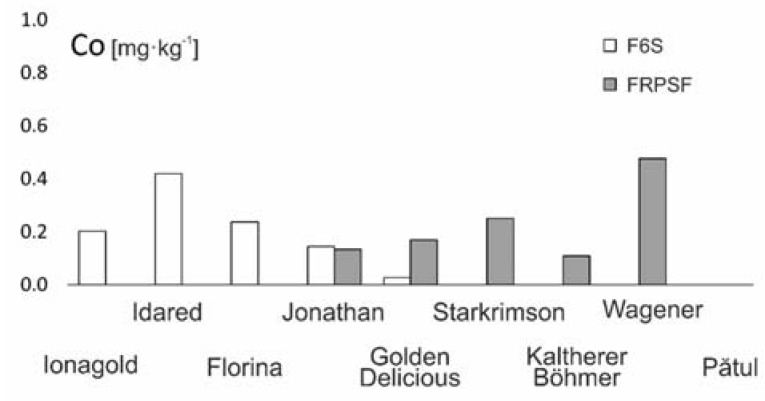
Co content variation according to apple varieties in FRPSF and F6S orchards. FRPSF—Fruit Research and Production Station of Fălticeni; F6S—Farm no.6 Sârca.

In this study, Co contents vary between 0.027 and 0.476 mg·kg^−1^, with higher contents being found for the Wagener and Idared varieties and lower contents being found for the Golden D. and Pătul varieties (Figure 16). At Fălticeni, Co is distributed relatively uniform. The only exception is the Wagener variety. The same areal distribution is valid for F6S, with the exception of the Idared variety.

There was no notable influence of adjacent traffic on Co contents in apples, and the higher contents in some varieties can be linked to an intrinsic Co accumulation capacity of the variety itself. Similar values are reported in other studies (Table 3).

## 4. Conclusions

In this study, trace element levels in nine different apple varieties were investigated. Average concentrations found are comparable with those reported in other countries.

Adjacent transportation activities did not seem to have a notable influence on trace element contents in apples, with the exception of toxic trace elements Pb and Cd. In the case of the two elements, higher concentrations were determined near the road at F6S, which has a high volume of car traffic. This proportional accumulation of toxic trace elements with the distance to the road at F6S is best visible in the case of Pb distribution map.

No influence of road vicinity on the trace elements distribution in apples was noticed at FRPSF, which lays near a county road, with low to very low car traffic volumes.

Three apple varieties have better accumulation capacities for dietary recommended trace elements such as Zn, Fe, and Cu. Good Fe and Zn sources are shown to be the Golden Delicious and Jonathan varieties. The Kaltherer Böhmer variety is an excellent source of Cu.

## Figures and Tables

**Table 1:** The average contents of water (%) and studied trace elements (mg·kg^−1^) in fresh fruit for the 9 analyzed apple varieties from FRPSF and F6S orchard. FRPSF—Fruit Research and Production Station of Fălticeni; F6S—Farm no.6 Sârca.

**Orchard**	**Apple Variety**	**No. of Samples**	**H_2_O Content**	**Zn**	**Cu**	**Fe**	**Mn**	**Ni**	**Cr**	**Co**	**Pb**	**Cd**
			%	mg·kg−1								
**Fălticeni (FRPSF)**	Golden Delicious	10	85.881	3.570	0.204	2.476	0.573	0.215	0.048	0.168	0.576	0.081
	Jonathan	4	86.095	2.587	0.273	1.725	0.545	0.122	0.101	0.133	0.797	0.088
	Kaltherer Böhmer	3	85.821	0.910	0.409	2.856	0.651	0.225	0.068	0.109	0.477	0.094
	Pătul	1	85.651	0.909	0.055	0.873	0.485	0.163	0.007	-	0.626	0.077
	Starkrimson	2	86.758	0.914	0.207	1.104	0.328	-	0.064	0.250	0.544	0.083
	Wagener	1	86.282	1.223	0.220	0.700	0.459	0.054	0.058	0.476	0.413	0.025
**Sârca (F6S)**	Golden Delicious	1	82.136	4.458	0.273	1.895	0.695	0.257	0.005	0.027	1.149	0.031
	Florina	4	83.265	3.255	0.239	0.927	0.470	0.168	0.041	0.237	0.683	0.111
	Idared	7	83.359	2.380	0.247	1.745	0.563	0.199	0.093	0.420	0.846	0.123
	Jonagold	4	84.136	1.046	0.149	1.349	0.469	0.131	0.036	0.202	0.875	0.111
	Jonathan	5	81.237	2.690	0.103	2.926	0.490	0.231	0.096	0.144	0.834	0.127
	* MAL			5	5						0.5	0.05

* MAL—The maximum permitted content thresholds in fresh fruit destined for sales and human nutrition established by Romanian national legislation [22,23].

**Table 2:** Correlation coefficients for trace elements content in apples from FRPSF and F6S orchards. FRPSF—Fruit Research and Production Station of Fălticeni; F6S—Farm no.6 Sârca.

**Element**	**Zn**	**Cu**	**Fe**	**Mn**	**Ni**	**Cr**	**Co**	**Pb**	**Cd**
Zn	1.00								
Cu	−0.16	1.00							
Fe	−0.16	0.33	1.00					FRPSF	
Mn	−0.01	0.22	0.24	1.00					
Ni	−0.09	0.08	0.49	0.17	1.00				
Cr	0.19	0.33	0.01	−0.06	−0.09	1.00			
Co	−0.24	−0.43	−0.22	−0.34	0.02	0.09	1.00		
Pb	−0.30	0.05	0.16	−0.10	−0.06	0.37	−0.10	1.00	
Cd	−0.01	0.29	0.02	−0.15	0.01	0.00	−0.48	0.22	1.00
**Element**	**Zn**	**Cu**	**Fe**	**Mn**	**Ni**	**Cr**	**Co**	**Pb**	**Cd**
Zn	1.00								
Cu	0.17	1.00							
Fe	0.22	0.14	1.00					F6S	
Mn	−0.04	0.26	−0.16	1.00					
Ni	0.17	0.03	0.26	−0.09	1.00				
Cr	−0.03	−0.25	0.03	−0.29	0.43	1.00			
Co	0.28	0.29	0.42	−0.28	0.13	0.02	1.00		
Pb	0.09	0.39	0.30	0.15	0.30	0.35	0.04	1.00	
Cd	−0.27	0.43	0.00	−0.05	0.01	0.05	0.35	0.39	1.00

**Table 3:** Trace elements content in apples from different studies

Country	Reference	Zn (mg·kg^−1^)	Cu (mg·kg^−1^)	Fe (mg·kg^−1^)	Mn (mg·kg^−1^)	Ni (mg·kg^−1^)	Cr (mg·kg^−1^)	Co (mg·kg^−1^)	Pb (mg·kg^−1^)	Cd (mg·kg^−1^)
Croatia	[33] *	0.873–5.8			0.04–1.7					
Greece	[5] *	1.1–10.3	0.87–5.37	10–150	2.0–9.0	0.05–0.7	1.5–2.6	0.01–0.1	0.01–0.46	0.01–6.85
	[34] *	9.9–10.8	12.3–13.6	17.63–19.96	3.1–4					
Libya	[35] *	2.34 ± 0.13	1.5 ± 0.1			1 ± 0.145		0.437 ± 0.062	0.2 ± 0.06	0.06 ± 0.03
Pakistan	[36] *	35.7 ± 2.3		172 ± 11	6.5 ± 0.26			0.052 ± 0.005		
Poland	[27] **	0.453	0.319			0.182			0.031	0.0057
	[28] **	0.181	0.24		0.307				0.0072	0.002
Portugal	[37] **	0.2705	0.6661	1.5	0.3539	0.0557				0.0143
Romania	[25] **		2.1–4.3							
	[14] **		0.25–0.8			0.48–0.54			0.08–0.21	≤0.04
	[13] *	1.93 ± 0.62	3.57 ± 1.05	3.68 ± 0.96	1.81 ± 0.58		<0.1		<0.01	<0.01
Serbia	[29] *	2.04 ± 0.005	4.21 ± 0.03			0.74 ± 0.025			0.63 ± 0.125	0.115 ± 0
	[9] *	2.0 ± 0.4–12.00 ± 0.02	5.12 ± 0.01–34.0 ± 0.2			0.64 ± 0.03–3.69 ± 0.06			0.84 ± 0.07–2.44 ± 0.06	0.135 ± 0.005–0.225 ± 0
	[38] *	0.39–2.24	0.76–2.73	1.9–11.41	0.81–3.22	0.02–0.89	≤0.57	≤0.01	≤0.29	
Turkey	[39] *	3.55 ± 0.34–4.06 ± 0.28	0.43 ± 0.06–1.13 ± 0.07	19.47 ± 2.67–21.25 ± 2.14	0.56 ± 0.04–0.91 ± 0.07	1.44 ± 0.19–2.43 ± 0.09	0.98 ± 0.21–1.36 ± 0.10	0.51 ± 0.08–0.78 ± 0.14	0.54 ± 0.18–0.67 ± 0.06	0.26 ± 0.05–0.54 ± 0.09
	[40] *	71.7 ± 1.8	5.2 ± 0.1	55.0 ± 0.1	38.6 ± 0.4					
U.S.A.	[41] *	1.4	5.5	9.3	4.3	<0.20	0.43	0.16		
Worldwide (synthesis)	[10] *	1.2	1.1	6	1.3	0.06	0.008	0.0003–0.0012	0.05–0.2	0.05
	[10] **	0.03	0.03	0.6–0.9	0.01–0.2	0.003–0.08	0.013	0.083–0.16	0.001	0.003–0.03
**This study**	**	0.909–4.458	0.055–0.409	0.700–2.926	0.328–0.695	0.054–0.257	0.005–0.101	0.027–0.476	0.413–1.149	0.025–0.127
	*	6.338–25.287	0.383–2.883	5.100–20.144	2.473–4.590	0.395–1.585	0.030–0.726	0.150–3.470	3.013–6.432	0.174–0.739

(* dried apple fruits; ** fresh apple fruits).
